# Neurosurgical application of pineal region tumor resection with 3D 4K exoscopy via infratentorial approach: a retrospective cohort study

**DOI:** 10.1097/JS9.0000000000000707

**Published:** 2023-09-27

**Authors:** Wei Hua, Xin Zhang, Qijun Wang, Tianming Qiu, Zixiao Yang, Xiaowen Wang, Hao Xu, Jinsen Zhang, Guo Yu, Minjie Fu, Liang Chen, Wei Zhu, Ying Mao

**Affiliations:** a Department of Neurosurgery, Huashan Hospital, Fudan University; b National Center for Neurological Disorders; c Shanghai Key Laboratory of Brain Function Restoration and Neural Regeneration; d Neurosurgical Institute of Fudan University; e Shanghai Clinical Medical Center of Neurosurgery, Shanghai, People’s Republic of China

**Keywords:** exoscope, hydrocephalus, infratentorial approach, pediatrics, pineal region tumor, third ventricle

## Abstract

**Background:**

The pineal region tumors are challenging for neurosurgeons and can lead to secondary hydrocephalus. The introduction of the exoscope has provided clinical interventions with high image quality and an ergonomic system for pineal region tumor operations. In this study, the authors describe the exoscopic approach used to facilitate the surgical resection of pineal region tumors and relieve hydrocephalus.

**Materials and methods:**

In this retrospective cohort study, we consecutively reviewed the clinical and radiological data of 25 patients with pineal region lesions who underwent three-dimensional exoscopic tumor resection at a single center.

**Results:**

The patient cohort consisted of 16 males and 9 females, with an average age of 34.6 years (range, 6–62 years; 8 cases aged ≤18). Pathological examination confirmed eight pineal gland tumors, four gliomas, nine germ cell neoplasms, two ependymomas, and two metastatic tumors. Preoperative hydrocephalus was present in 23 patients. Prior to tumor resection, external ventricular drainage (EVD) with Ommaya reservoir implantation was performed in 17 patients. Two patients received preoperative endoscopic third ventriculostomy (ETV), and five patients received a ventriculoperitoneal (VP) shunt, including one who received both procedures. Gross total resection was achieved in 19 patients (76%) in the ‘head-up’ park bench position using the exoscope. Eight patients (31.6%) with third ventricle invasion received subtotal resection, mainly in glioma cases, which was higher than those without invasion (0%), but not statistically significant (*P*=0.278, Fisher’s exact test). No new neurological dysfunction was observed after surgery. Two patients (8%) developed intracranial and pulmonary infections, and two patients (8%) suffered from pneumothorax. Hydrocephalus was significantly relieved in all patients postoperatively, and four patients with relapse hydrocephalus were cured during the long-term follow-up. Postoperative adjuvant management was recommended for indicated patients, and a mean follow-up of 24.8±14.3 months showed a satisfied outcome.

**Conclusions:**

The exoscope is a useful tool for pineal region tumor resection and hydrocephalus relief, particularly with posterior third ventricle invasion, as total resection could be achieved without obvious complication. The special superiority of the exoscope for the indicated pineal region tumors should be highlighted.

## Introduction

Highlights

- The 3D 4K exoscope is an effective tool for pineal region tumor resection with the advantage of combination with a microscope and endoscope.
- Gross total resection was achieved in 76% of patients with the exoscope in the ʻhead-upʼ park bench position.
- The exoscope may be particularly useful in cases of pineal region tumor with posterior third ventricle invasion.

The use of microscopes, endoscopes, and ventriculoscopes has revolutionized neurosurgical illumination and techniques, providing significant benefits to both neurosurgeons and patients. In 2008, the high-definition exoscope (HDXO-SCOPE) system was introduced as a new tool for neurosurgical procedures^1^, and it has since been widely used in both cranial and spinal surgeries^2–7^. Technological developments have now updated it to a 3-dimensional, high-definition (3D, 4K) display system^8,9^. Despite its potential advantages, the application of an exoscope in neurosurgery has not been extensively discussed, as most neurosurgeons are trained and accustomed to using a microscope and endoscopes.

The pineal region presents a complex anatomy and a wide range of pathologies, making it a challenging area for neurosurgeons. Although various surgical approaches have been introduced to access the posterior third ventricle, such as the supracerebellar infratentorial (SCIT) approach with a microscope or endoscope, and the suboccipital transtentorial approach (Poppen approach) with a microscope^10–12^, these approaches have their limitations. Endoscopic resection of pineal region tumors is typically limited to small and unvascularized lesions^13^, and requires extra efforts to stabilize the facility, while microscopic resection often involves nonergonomic postures^4,6,8^. The exoscope can compensate for these limitations with its fine-tuned, high-definition 3D image and extended focal depth, which is essential for deep-seated pineal region surgeries^6,14^. Studies have shown that even 2D exoscopes can achieve satisfactory resection of giant vestibular schwannoma, equivalent to surgical microscopes^15^. Moreover, 3D exoscopes can offer superior advantages over 2D exoscopes for beginners and maintain their excellence even when wearing protective suits during the COVID-19 pandemic^16,17^. The exoscopic operation is completed by viewing the monitor, allowing operators to avoid unergonomic postures during pineal lesion resection and ensuring delicate operations^17^. However, to date, there has been no large-scale cohort study evaluating the surgical outcomes of patients with pineal region lesions who have undergone exoscopic surgery.

In this retrospective cohort study, we investigated the use of exoscopes in the resection of indicated pineal region tumors and evaluated their benefits and limitations. We reviewed the experience of 25 consecutive cases to provide insights into the potential of exoscopic resection techniques and enable the next generation of digital image-based neurosurgery, allowing safe and effective removal of more complex lesions in the pineal region.

## Materials and methods

### Study design and patient selection

This study involved a cohort of patients with pineal region lesions who underwent exoscopic surgery. A total of 25 patients with pineal region lesions were retrospectively enrolled at our center between April 2018 and November 2021. All patients underwent exoscopic resection of the pineal region tumors *via* the SCIT approach. In cases where patients presented with symptoms or radiological indications of hydrocephalus, a preoperative Ommaya reservoir was implanted to address potential high intracranial pressure crises. VP shunt or ETV were also performed, if permanent hydrocephalus developed. Patients who underwent surgical resection solely with a microscope or endoscope were excluded from the study. Preoperative medical records and radiological images were meticulously reviewed.

This cohort research was approved by the Institutional Review Board of the hospital and was conducted in accordance with the Helsinki Declaration. Written informed consent was obtained from all patients. This work was reported in line with the Strengthening the reporting of cohort studies in surgery (STROCSS) criteria^18^ (Supplemental Digital Content 1, http://links.lww.com/JS9/B101) and was retrospectively registered at Research Registry.com.

### Exoscope specifications

The VITOM 3D exoscope (KARL STORZ SE & Co. KG) was used in all procedures. The system consists of an IMAGE1 S CONNECT module, an IMAGE1 S D3-LINK module, a 4K (4096×2160 pixels) sensor, a POWER LED light source, and a light cable. The image is displayed in full high-definition (HD, 1080p resolution) on a 32-inch active 3D screen, and 3D glasses are required for extensive exoscopic visualization.

### Exoscopic operative technique for pineal region tumor resection

Patients were placed in a modified ʻhead-upʼ park bench position (the upper body is elevated, and the head is slightly extended instead of anteflexion) to gain enough auto-retraction by gravity and counter the difficulty of the semi-sitting position. Then the head was secured in place with a head clamp (Mayfield). Neuronavigation was registered using either the Medtronic System (Medtronic, Inc.) or the Stryker Navigation System (Stryker, Kalamazoo). A midline suboccipital incision was made with the superior limb curved toward the side of the lesion. The cerebrospinal fluid (CSF) of the cisterna magna was slowly released after opening the dura to decrease the intracranial pressure. Through the combination of gravity assistance and CSF release, the corridor between the cerebellum and tentorium was opened without any retractor.

Through the infratentorial corridor, the arachnoid membrane was gently incised and peeled off to visualize the pineal region. The bilateral feeding arteries originating from the branches of the posterior cerebral artery or superior cerebellar artery were cauterized and transected before circumferential debulking. The debulking could be easily performed with an ultrasonic dissector if necessary. Intraoperative pathology was routinely performed. A precise plane between the neoplasm and normal brain tissue would be carefully identified using the exoscope with high-definition images. After tumor resection, the surgeon would have a thorough view of the third ventricle to verify the extent of resection and perform necessary hemostasis. The dura was closed, and the bone flap was consequently plated in place.

### Postoperative management and follow-up

Postoperative management routinely included prophylactic antibiotics (cefuroxime sodium for injection, 0.75 g, 1.5 g bid intravenous infusion, Salubris Pharmaceuticals Co., Ltd., catalog Number 86900553000352), antiseizure prescriptions (sodium valproate sustained-release tablets, 0.5 g, 0.5 g bid orally, Sanofi, catalog number 86978820000756) and dehydration drugs (20% mannitol injection, 250 ml, 250 ml q8h intravenous infusion, Baxter International Inc., catalog Number 86900628000430). Neurological and radiological examinations were routinely performed. Postoperative MR scans were carried out within 72 h to determine the extent of resection. Postoperative complications were carefully managed, and adjuvant radiotherapy and/or chemotherapy were administered according to the pathology and guidelines. All patients were scheduled for regular follow-up.

### Clinical data acquisition and outcome assessment

Essential clinical data were obtained, including age, sex, preoperative symptoms, tumor pathology, tumor size, relation to the third ventricle, hydrocephalus status, extent of resection, length of operation, and adjuvant therapy. For patients diagnosed with germ cell tumors, we also collected preoperative and postoperative AFP (α-fetoprotein) and β-HCG (β-human chorionic gonadotropin) levels.

Both short-term and long-term outcomes were evaluated. Short-term complications, hydrocephalus status, and the corresponding treatment were carefully documented. Survival data were also collected for analysis.

### Statistical analysis

Statistical analysis was performed using GraphPad Prism 9.0 software (GraphPad Software). To assess the normality of continuous variables, the Shapiro–Wilk test was initially conducted. For comparisons between two samples, the unpaired *t*-test was utilized if the data followed a normal distribution. In cases where the data did not conform to a normal distribution, the Whitney–Mann *U* test was applied. For categorical variables, either a *χ* ^2^ analysis or Fisher’s exact test was used, as appropriate. All statistical tests were two-tailed, and *P*-values less than 0.05 were considered statistically significant.

## Results

### Clinical manifestation of 25 patients with pineal region tumors

The clinical features of the 16 male and 9 female patients with pineal region tumors are summarized in Table 1. The mean age was 34.6 years old (range from 6 to 62), and 8 cases aged less than or equal to 18 provided an insight into exoscope utilization in pediatrics. While one patient was diagnosed incidentally, the other 24 patients reported multiple onset symptoms, including headache (56.0%), dizziness (52.0%), visual disturbance (50.0%), vomiting (40.0%), unsteady walking gait (28.0%), and Parinaud syndrome (16.0%). EVD with Ommaya reservoir implantation was performed in 17 patients with preoperative hydrocephalus. Two patients received preoperative ETV, and five patients received VP shunt treatment, including one who underwent both procedures. Six patients had elevated AFP levels (four with mixed germ cell tumors and two with teratomas), and two patients had elevated β-HCG levels (one patient with a mixed germ cell tumor and one patient with a mature teratoma).

**Table 1:** Clinical features and management of the 25 patients with pineal region tumors.

Age (years)/ Sex	Symptoms	Pathology	Tumor size (cm)	Invading 3rd ventricle	Hydrocephalus and treatment	Operation date	Length of operation(h)	EOR	Complications	Adjuvant therapy	Follow-up
25/M	Double vision, dizziness	Germinoma	2.3×1.2×1.3	Yes	Yes/Ommaya	2018/4/26	5.5	GTR	Later ETV^a^	Rx	SD (49 months)
14/M	Dizziness, vomiting	Mixed germ cell tumor	4.0×3.0×1.8	Yes	Yes/VP	2018/6/21	8	GTR	Unsteady gait	Rx	SD (47 months)
59/F	Unsteady walk	Pinealocytoma (grade 2)	2.4×1.7×1.2	No	Yes/Ommaya	2018/7/3	5.75	GTR	None	–	SD (47 months)
59/M	Headache, dizziness, blurred vision	Metastasis (Lung)	3.4×3.2×2.1	Yes	Yes/Ommaya	2018/8/22	6.67	GTR	Pneumothorax	Chemo	SD (45 months)
19/M	Headache, dizziness, unstable gait, blurred vision	Germinoma	2.7×2.3×2.1	Yes	Yes/Ommaya	2018/10/16	5.33	GTR	None	Rx	SD (43 months)
22/F	Headache, vomiting, blurred vision, unstable gait	Pilocytic astrocytoma (grade 1)	1.7×1.3×1.4	Yes	Yes/Ommaya	2019/2/12	5	STR	Subtentorial hydroma, ETV^a^	–	SD (39 months)
62/F	Faint, vomiting	Pinealocytoma (grade 1)	1.9×0.8×0.6	Yes	Yes/Ommaya	2019/4/23	5	GTR	None	–	SD (37 months)
18/M	Blurred vision, headache, dizziness, vomiting	Mixed germ cell tumor	2.8×2×1.6	Yes	Yes/Ommaya	2019/6/21	5	GTR	None	Chemo without Rx	Dead (6 months)
13/M	Strabismus, tremor, weakness, sexual precocity	Matured teratoma	4.8×5.2×4.7	Yes	Yes/Ommaya	2019/7/18	5.75	GTR	None	–	SD (34 months)
14/M	Dizziness, unsteady walk	Ependymoma (grade 2)	2.8×2.2×1.5	No	Yes/Ommaya	2019/8/22	7.5	GTR	Scalp hydrops	-	SD (33 months)
6/M	Vomiting, blurred vision	Mixed germ cell tumor	2.8×2.5×2.2	Yes	Yes/Ommaya	2020/2/17	3.75	GTR	None	Rx	SD (27 months)
29/F	Dizziness, headache, vomiting, hyperopia	Glioblastoma	3.6×3.2×3.3	Yes	Yes/ETV/VP	2020/2/19	5.75	STR	None	Rx+Chemo	Dead (12 months)
19/M	Vision decline	Low-grade glioma (grade 1)	1.2×0.9×0.8	Yes	Yes/VP	2020/4/30	5.33	STR	None	–	SD (25 months)
60/F	Headache	Pinealocytoma	3.3×2.2×1.2	Yes	Yes/Ommaya	2020/5/15	5	GTR	None	–	SD (24 months)
55/F	None	Metastasis (Breast)	4.5×3.1×1.5	No	No/No	2020/7/23	6	GTR	None	Rx+Chemo	Dead (9 months)
59/F	Headache, vomiting, unstable walk, blurred vision	Glioblastoma	1.8×1.2×0.9	Yes	Yes/VP	2020/9/15	8	STR	Pneumothorax	Rx+Chemo	SD (20 months)
57/M	Unstable gait	Papillary tumor (grade 2)	2.3×2.0×1.5	Yes	Yes/Ommaya	2020/11/9	4.67	GTR	None	Chemo	SD (19 months)
52/M	Dizziness, headache	Pineal parenchymal tumor (grade 3)	1.4×1.4×1.2	Yes	Yes/Ommaya	2020/12/3	6	STR	None	Rx+Chemo	SD (18 months)
46/M	Fatigue	Pineal parenchymal tumor (grade 3)	2.4×2.4×2.2	Yes	Yes/Ommaya	2020/12/7	6	STR	Apathy, later ETV^a^	Rx+Chemo	SD (18 months)
14/M	Headache, blurred vision, mental decline	Mixed germ cell tumor	3.1×2.5×2.7	Yes	No/No	2021/3/4	5.5	GTR	None	Rx	SD (15 months)
40/M	Headache	Ependymoma (grade 3)	1.3×1.1×1.0	No	Yes/Ommaya	2021/4/8	4.75	GTR	None	Rx+Chemo	SD (14 months)
52/F	Headache, vomiting	Pineal parenchymal tumor (grade 2)	2.9×2.3×2.8	No	Yes/VP	2021/5/31	5	GTR	None	–	SD (12 months)
8/M	Headache, vomiting, double vision	Immature teratoma	4.8×3.7×3.4	No	Yes/ETV	2021/6/17	5	GTR	Brain infection	Rx	SD (11 months)
12/M	Blurred vision, unsteady walk	Matured teratoma	8.2×5.4×2.4	Yes	Yes/Ommaya	2021/7/12	15	GTR	Later VP^a^	–	SD (10 months)
50/F	Dizziness, headache	Pineal parenchymal tumor (grade 2)	1.4×1.1×0.8	Yes	Yes/Ommaya	2021/11/19	7	GTR	Lung infection	–	SD (6 months)

EOR, extent of resection; GTR, gross total resection; STR, subtotal resection; ETV, endoscopic third ventriculostomy; VP, ventriculoperitoneal shunt; Rx, X-ray radiotherapy; Chemo, chemotherapy; SD, stable.

a Relapse hydrocephalus after discharge.

**Figure 1:**
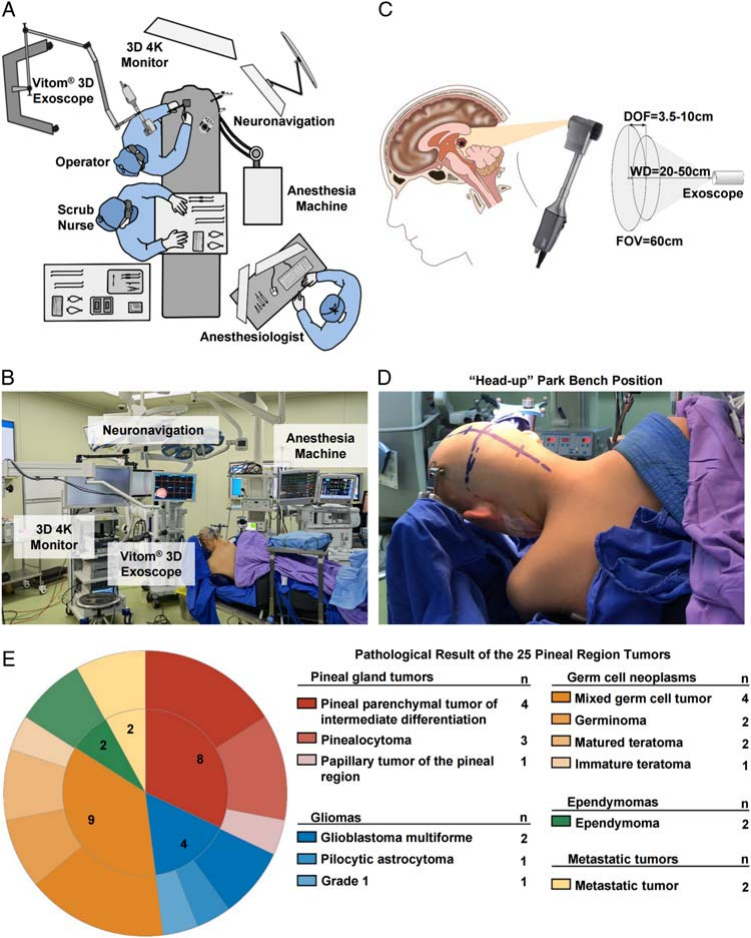
A: The setup of the operation theater for the exoscopic pineal region tumor resection. B: The real-life image of the operation theater. C: The infratentorial corridor used in our series and the parameters of the VITOM 3D exoscope used in all procedures. D: The ‘head-up’ park bench position. E: Pathological composition of the 25 pineal region tumors in this cohort. DOF, depth of field; FOV, field of view; WD, working distance.

**Figure 2:**
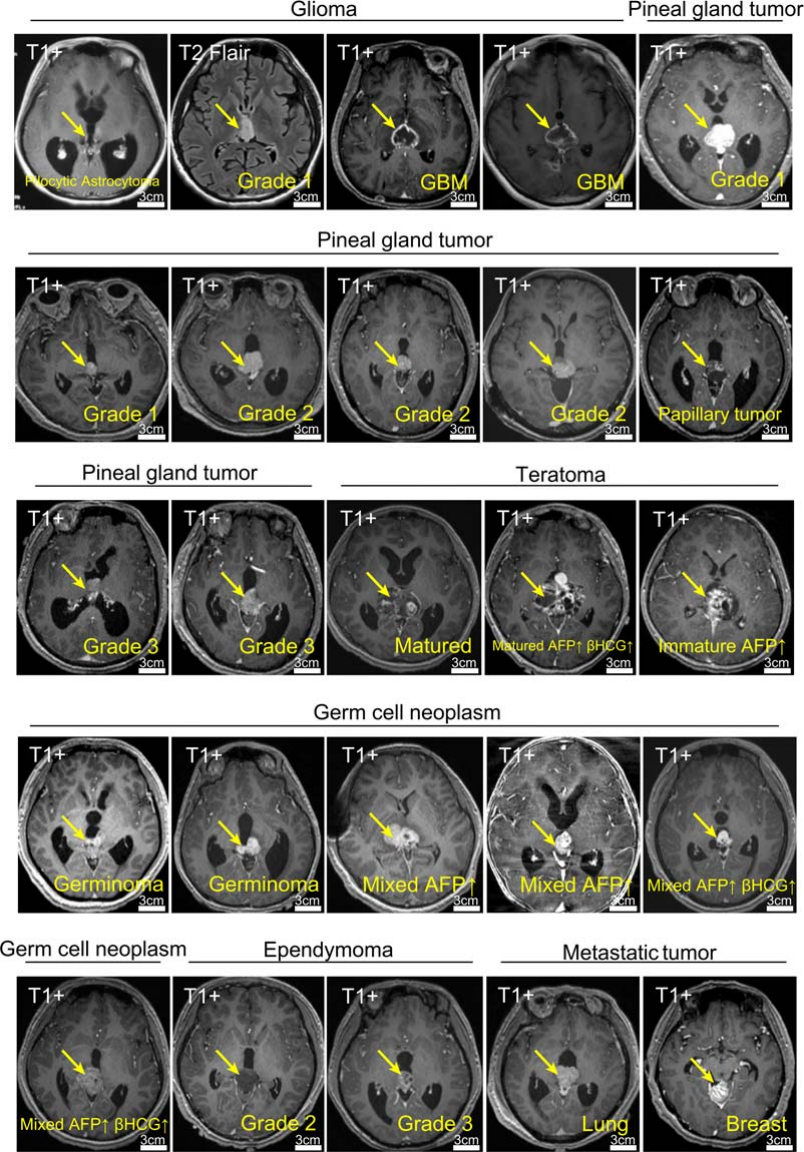
The category of 25 pineal region tumors with annotation of pathology, grade, and some laboratory information, and tumor marked with arrowheads. The scale bars are shown in the MR images. AFP, α-fetoproteinand; β-HCG, β-human chorionic gonadotropin.

In this cohort, the mean tumor volumes were 10.9±15.7 cm^3^, and the diameters of 14 tumors (56.0%) were larger than 2.5 cm. Nineteen tumors invaded the third ventricle. Pathological examination confirmed eight pineal gland tumors (including four pineal parenchymal tumors of intermediate differentiation, three pineocytomas, and one papillary tumor of the pineal region), four gliomas [including two glioblastomas (GBM), one pilocytic astrocytoma, and one low-grade glioma], nine germ cell neoplasms (four mixed germ cell tumors, two germinomas, two mature teratomas and one immature teratoma), two ependymomas and two metastatic tumors (Figs. 1E, and 2).

### The exoscope could provide an ergonomic style for pineal region tumor resection

The operation theater could be organized to apply the exoscope, as shown in Figure 1A and B. All 25 cases were operated on with an exoscope in a ʻhead-upʼ park bench position to achieve gravity-retracted assistance (Fig. 1B and D). The 3D-4K exoscope provided a 60 cm field of view, a 3.5–10 cm depth of field, a 20–50 cm working distance, and high 4K image quality (Fig. 1C). The magnification of the exoscope could be adjusted 8–30× on a 32-inch monitor. In addition, all the participants in the theater could benefit from an ergonomic condition (Supplementary Fig. 1, Supplemental Digital Content 2, http://links.lww.com/JS9/B102) and didactic operational aims, and the exoscope could operate in conjunction with the intraoperative navigational system and other assistance (Fig. 1B). In our series, the exoscope was applied in all cases, and only one case was switched to the microscope due to the time-consuming adjustment of the focus length.

The mean duration of the operation was 6.1±2.1 h. We categorized the 25 cases into two groups based on the chronological surgical dates: 2018–2019 (*n*=10) and 2020–2021 (*n*=15). No significant differences were observed in the operating time (5.6 vs. 5.5 h, *P*=0.675) and the maximal diameter of the tumors (2.8 vs. 2.8, *P*=0.803) between the two groups (Table 1). All patients were illustrated in Figure 2 with annotations. Gross total resection (GTR) was achieved in 19 patients (76.0%), which is comparable to the reported GTR rates in the literature for microscopic surgery of pineal region tumors. Among the tumors with invasion into the third ventricle, the GTR rate was 68.4%, which was lower than that in cases without invasion (100%); however, this difference was not statistically significant (*P*=0.278, Fisher’s exact test). In the eight pediatric cases, all patients achieved GTR. Subtotal resection was performed when achieving GTR was not feasible.

### Postoperative management of hydrocephalus and complications

A significant decline of AFP level could be observed one week after resection in the patient with a mixed germ cell tumor, while β-HCG level soon dropped back to normal postoperatively (not shown). Hydrocephalus was significantly relieved among all patients after surgeries. Four patients suffered from relapsed hydrocephalus within 3 months after discharge and were treated with an ETV or VP shunt afterwards. Tentorial subdural hygroma was observed in one case, which was relieved after ETV. Scalp hydrops was seen in one pediatric patient (Table 1).

Intracranial and pulmonary infections were seen in two patients (8%), which were all cured by antibiotics. Two patients (8%) experienced pneumothorax. Closed thoracic cavity drainage was administered for these two patients, and the symptoms were quickly relieved. One case with upward gaze paralysis and one with blurred vision remained the same after the surgery. No new neurological dysfunction, venous infarction, or air embolization was observed.

### Exoscopic resection could improve the prognosis for the pineal region tumors

Although the long-term clinical outcome still relies on the tumor malignancy and adjuvant therapy, exoscopic resection could dramatically increase the GTR ratio and relieve hydrocephalus, thus improving the quality of life. In this cohort, postoperative adjuvant treatment was managed according to the specific pathology of each patient. Patients with gliomas (except pilocytic astrocytoma) would receive concomitant radio-chemotherapy. Patients diagnosed with germ cell neoplasms would undergo radiotherapy alone or combined with chemotherapy. One patient with a mixed germ cell tumor only received chemotherapy without radiation for economic reasons. The mean follow-up time was 24.8±14.3 months, and 22 out of the 25 patients had no recurrence, including one with GBM (20 m) and one with brain metastasis (45 m). One patient with brain metastasis from breast cancer died 9 months after the resection, and one patient with GBM died 12 months after the surgery. The patient with a mixed germ cell tumor without postoperative radiotherapy died 6 months after the surgery due to distal recurrence, while other patients with germ cell tumors achieved a satisfactory clinical outcome (Table 1).

### Case illustration

#### Case one

**Figure 3:**
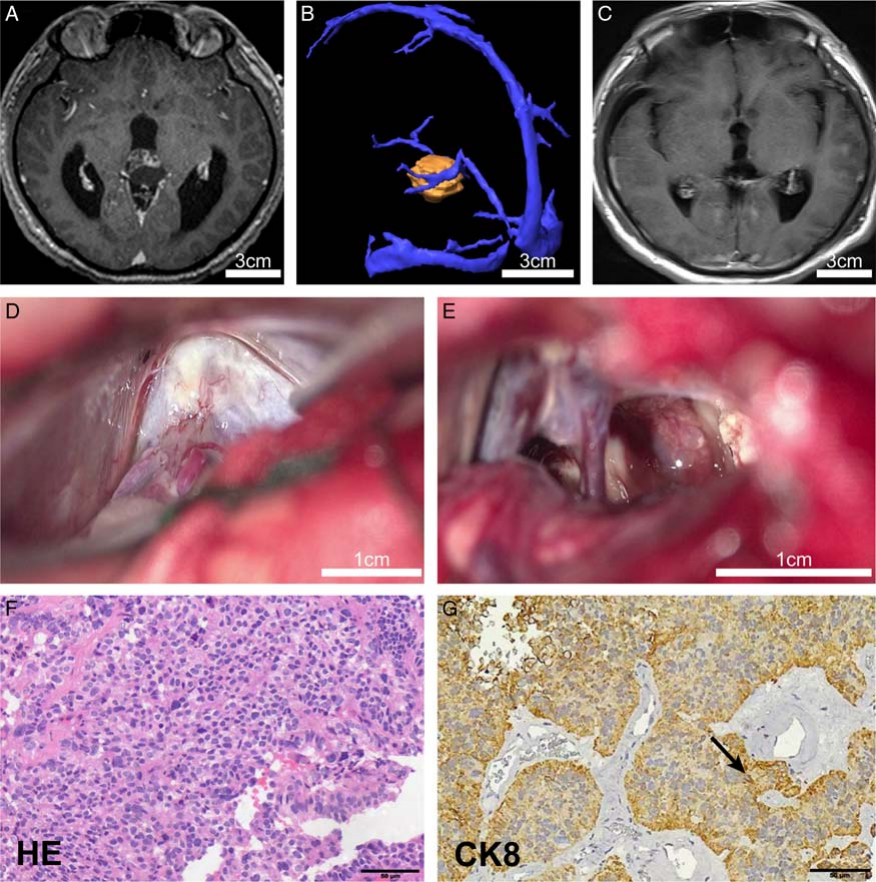
The illustrated case 1 of pineal papillary tumor resection with exoscope. A: MRI image shows the lesion located at the pineal region, with heterogeneous enhancement after gadolinium injection. B: The MRV reveals a close association of the tumor with the inferior sagittal sinus. C: Postoperative MR image confirms gross total resection. D, E: The tumor is resected via the infratentorial corridor using the exoscope. F: hematoxylin-eosin (HE) staining is supportive for pineal papillary tumor (×400). G: Diffused tumor cells stained positive for cytokeratin 8 (CK8, ×400, arrowhead).

A 57-year-old man presented with a three-week history of an unstable gait. An MRI examination revealed a pineal region tumor with heterogeneous enhancement and obvious hydrocephalus (Fig. 3A). The MRV showed that the tumor was closed related to the inferior sagittal sinus (Fig. 3B). Blood AFP and β-HCG levels were not elevated. An Ommaya reservoir was implanted first, followed by neurosurgical resection via the SCIT approach with navigation. The quadrigeminal and ambient cisterns (Fig. 3D), and the posterior portion of the third ventricle could be visualized panoramically with the exoscope (Fig. 3E). A clear plane between the tumor and veins was identified, as was shown in the video (Video 1 in Supplementary, Supplemental Digital Content 3, http://links.lww.com/JS9/B103). Postoperative images confirmed GTR, and hydrocephalus was relieved (Fig. 3C). Intraoperative frozen section indicated a papillary tumor, which was confirmed by the final pathology (Grade 2, Fig. 3F, G). Chemotherapy with temozolomide was recommended, and no recurrence was observed 18 months after surgery (Supplementary Fig. 2, Supplemental Digital Content 2, http://links.lww.com/JS9/B102).

#### Case two

**Figure 4:**
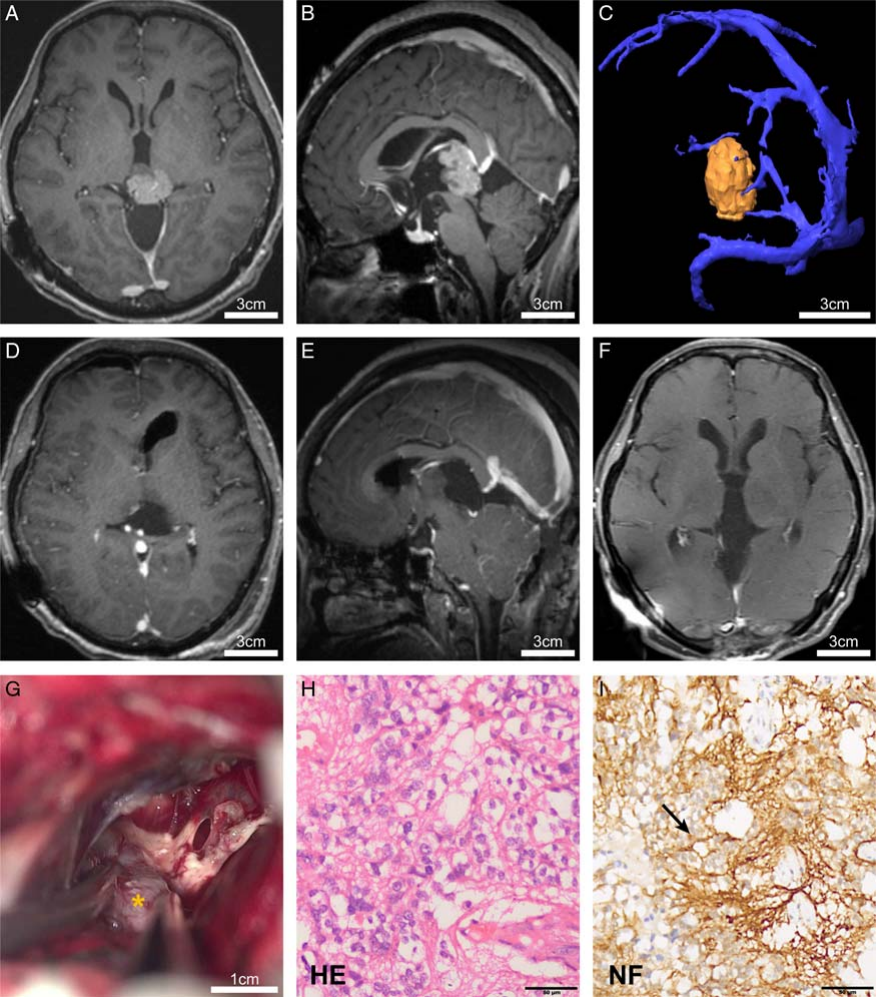
The illustrated case 2 of pineal parenchymal tumor resection with exoscope. A, B: MRI images indicate that the lesion is located in the pineal region, heterogeneously enhanced after gadolinium injection. C: The MRV reveals a close relationship of the tumor with the vein of Galen. D, E: Postoperative images confirm gross total resection. F: Nine-month follow-up MRI shows no recurrence of the tumor. G: The tumor is resected via the infratentorial corridor using the exoscope (*Tumor). H: HE staining is supportive for pineal parenchymal tumor (×400). I: The immunohistochemistry staining is positive for neurofilament (NF, ×400, arrowhead).

A 52-year-old woman complained of headaches and vomiting for 3 months. The patient underwent VP shunt surgery in a local hospital. MRI showed a lesion at the posterior third ventricle with hyperintensity on T1 contrast images (Fig. 4A, B). The tumor was found to be closely related to the vein of Galen on MRV (Fig. 4C). Both blood AFP and β-HCG levels were normal. Neurosurgical resection was performed via the SCIT approach, and through the infratentorial corridor, the posterior third ventricle could be visualized clearly with the exoscope, and a clear plane between the tumor and the vein of Galen, brainstem, and thalamus was identified to achieve GTR, as shown in the video (Fig. 4G, Video 2 in Supplementary, Supplemental Digital Content 4, http://links.lww.com/JS9/B104). Intraoperative frozen pathology indicated a pineal parenchymal tumor. Postoperative images confirmed GTR (Fig. 4D, E), and final pathology demonstrated a pineal parenchymal tumor of intermediate differentiation (Grade 2, Fig. 4H, I). After surgery, the patient’s symptoms were relieved, and no radio-chemotherapy was administrated. No recurrence was found 9 months after surgery (Fig. 4F).

## Discussion

Surgical resection for pineal region tumors has been challenging but necessary, as radical resection can lead to better clinical outcomes for both benign and malignant tumors^19^, and can relieve the symptoms of hydrocephalus^20^. Additionally, obtaining sufficient surgical tissues can support integrated diagnosis and avoid misdiagnosis of mixed germ cell tumors, which can be facilitated with an appropriate neurosurgical approach and equipment^20^. Based on our experience, we believe that the exoscope has demonstrated superiority for indicated pineal region tumors, while offering only congruency for other neurosurgical cases. In this study, we present a cohort of 25 pineal region tumor resections using an exoscope, with a focus on its advantages for deep, large, and indicated posterior third ventricle or pineal region tumors.

The utilization of the exoscope in brain tumor resection has been prevalent since its introduction^1^. Our review of the literature showed that in certain aspects, the exoscope was superior to the operative microscope and neuroendoscope, including a wider field of view, 3D visual perception, reasonable size, less cumbersome and good ergonomics^2,5–7,9,21^. The magnification of the exoscope could be adjusted between 8–30× on a 32-inch monitor, which is at least comparable to the conventional microscope^22^. With these features, the quadrigeminal and ambient cisterns, as well as the posterior third ventricle, could be viewed panoramically through the infratentorial corridor. In our case series, these unique features of the exoscope allowed for a reasonable rate of GTR (76%) for larger tumor volumes compared to previous reports of microscopic pineal surgery (57.2–88.9%)^10,11,23,24^. However, the rate of GTR was lower than that reported for purely endoscopic or endoscopic-assisted surgery (90–100%)^23,25^. This difference may be attributed to patient selection, as tumors with invasion into the third ventricle tended to achieve subtotal resection in our current cohort. Simultaneously, the prompt relief of hydrocephalus, a significant decrease in AFP and β-HCG levels, and a favorable long-term prognosis demonstrated the superior surgical outcome of exoscopic resection.

Apart from tumor resection, other clinical utilizations of exoscopes have been carried out. The implementation of an exoscope with the mandatory personal protective equipment in emergency operations during the COVID-19 pandemic has been reported^17^. Compared with the microscope, the 3D exoscopes would be more compatible with the protective precautions, including the face shields, goggles, and coveralls. In addition, microvascular anastomosis in latex vessel models under exoscopic assistance was put into practice by Pinto *et al.*, showing the great potential of the exoscope in the clinically educational field^26^. As the exoscope can provide high-quality visualization to residents and fellows, it is a good opportunity for them to engage with a visual perception of the surgical fields, anatomic details, and practical techniques in a feasible and surgeon-oriented way.

Since there is no standardized approach to pineal region tumor resection, the use of the exoscope system has become increasingly common in these surgeries since its introduction^1,6^. In comparison to microscopic surgery, the exoscope offers several advantages. Firstly, it eliminates the need for frequent focus adjustments, allowing for a consistently clear view throughout the procedure. Additionally, the ability to swiftly and effortlessly switch between micro and macro views is enhanced with the exoscope. Furthermore, the slim camera design provides surgeons with a longer working distance, enabling smoother utilization of equipment^27^.

However, its drawbacks, including insufficient resolution in deep brain surgeries due to a distant light source, a prolonged training period for relevant surgical participants, a learning curve for eye-hand coordination without direct observation of the surgical site, and 3D goggles restrictions, hinder its widespread adoption^9,28^. Therefore, the most appropriate indication for the exoscope still requires further investigation, as it is not simply a substitute for a microscope or endoscope.

During our four-year practice utilizing the exoscope, we did not observe the anticipated learning curve. Furthermore, the average operating time in this study, despite the intricate nature of pineal region surgeries, was not excessively lengthy when compared to microscopic procedures. This suggests that experienced neurosurgeons can quickly adapt to and proficiently manipulate the 3D exoscope.

In pediatric cases, mixed germ cell tumors are the most frequently encountered pathological subtype, and their deep location makes their removal particularly challenging^2^. However, in our pediatric procedures, the combination of the left ʻhead-upʼ park bench position and adequate illumination enabled successful tumor resection. The modified park bench position has been shown to reduce the incidence of venous air embolism compared to the traditional sitting position^29^. Similar to the sitting position, the modified position uses gravity to separate the tumor from the veins, facilitating tumor dissection without the need for retractors.

Most patients with pineal region tumors present with obstructive hydrocephalus, which can affect the surgical exposure and long-term clinical outcome^20^. There are several ways to manage hydrocephalus. EVD with Ommaya reservoir implantation is highly recommended in our experience, as it can rapidly relieve hydrocephalus and reduce intraoperative intracranial pressure, allowing more time for preoperative planning. Additionally, the Ommaya reservoir provides greater flexibility in controlling ventricular volume for satisfactory tumor manipulation compared to VP shunts and ETV^20^. ETV is particularly suitable for cases requiring biopsy, while VP shunts should be avoided if possible^20^. Although tumor resection can restore CSF circulation, hydrocephalus can progress in 12–81% of cases in the long-term^30^. In our study, hydrocephalus was promptly relieved after surgery in all patients, but four patients experienced relapsed hydrocephalus within 3 months after discharge, and three of them had undergone subtotal resection. Partial resection and tumor recurrence are the most common reasons for progressive or relapsed hydrocephalus^30^. Therefore, GTR and the opening of the third ventricle simultaneously could eliminate obstructive hydrocephalus in the long-term^20^.

Among the 25 cases, six patients had elevated levels of AFP, with four being diagnosed with mixed germ cell tumors and two with teratomas. β-HCG was elevated in two patients, one with a mixed germ cell tumor and one with a mature teratoma. An elevated level of AFP in serum or CSF is often seen in endodermal sinus tumors, embryonal carcinomas, or immature teratomas, while the presence of β-HCG suggests choriocarcinomas, embryonal carcinomas, or germinomas^31^. Our findings were generally in agreement with these known associations, but it should be noted that a few patients with germinomas or mature teratomas in our cohort had normal levels of β-HCG and AFP. Therefore, the absence of germ cell markers needs to be carefully interpreted to rule out the presence of these tumor types.

The current molecular subgrouping of the pineal and germ cell tumors is updating our understanding of their pathogenesis. Studies have identified important genetic alterations in pineal gland tumors, such as the *DICER1* and *DROSHA* mutations^32^. In addition, *CCND2*, *PRDM14*, and *RB1* were found to be significantly crucial in the oncogenesis of intracranial germ cell tumors^33^. Hopefully, these new insights will lead to a more precise diagnosis and innovative treatment concepts for pineal region tumors.

Some limitations of the exoscope still remain, such as the learning curve required to adapt to the eye-hand coordination and the risk of 3D-goggle-related eyestrain^6^. However, based on our experience, the exoscope is at least as effective as the operational microscope and endoscope in performing pineal region tumor resections. It is important to note that surgical management remains the preferred treatment for pineal region tumors and the secondary hydrocephalus^19,20^. With this in mind, the exoscope is certainly an option worth considering for posterior third ventricle tumors, as it can enable total resection for these challenging tumors compared to the endoscopes and microscopes.

## Conclusions

Overall, our experience of 25 consecutive cases highlights the special advantages of using an exoscope for pineal region tumor resection. The exoscope has demonstrated a great ability to facilitate total resection of pineal region tumors invading the posterior third ventricle and relieve hydrocephalus with minimal complications. Despite its limitations, the exoscope remains a valuable option for safely and effectively accessing and removing complex lesions in the pineal region.

## Ethical approval

The clinical study complied with the provisions of the Declaration of Helsinki. The study was approved by the Ethics Committee of Huashan Hospital (reference number 2019-586).

## Consent

Written informed consent was obtained from the patient for publication. A copy of the written consent is available for review by the Editor-in-Chief of this journal on request.

## Sources of funding

The authors would like to express their gratitude to the CAMS Innovation Fund for Medical Sciences (CIFMS, 2019-I2M-5-008) and Shanghai Development and Reform Commission (2018SHZDZX01) for their support.

## Author contribution

W.H., X.Z., and Q.W.: data collection, data analysis and interpretation, writing the paper; T.Q., Z.Y., X.W., H.X., J.Z., G.Y., and M.F.: patient selection and data collection; L.C., W.Z., and Y.M.: study design, writing the paper. All authors approved the submitted version of the manuscript.

## Conflicts of interest disclosure

The authors declare that they have no conflicts of interest.

## Research registration unique identifying number (UIN)

1. Name of the registry: http://www.researchregistry.com.
2. Unique identifying number or registration ID: researchregistry8934.
3. Hyperlink to your specific registration (must be publicly accessible and will be checked): https://www.researchregistry.com/browse-theregistry#home/registrationdetails/6450e63914b1840028360656/.

## Guarantor

Ying Mao and Wei Zhu.

## Provenance and peer review

Not commissioned, externally peer-reviewed.

## Data availability statement

The data underlying this article will be shared on reasonable request to the corresponding author (W.Z. and Y.M.). E-mail: maoying@fudan.edu.cn drzhuwei@fudan.edu.cn.

## Supplementary Material

SUPPLEMENTARY MATERIAL
