# Supplementary material for: Neurosurgical application of pineal region tumor resection with 3D 4K exoscopy via infratentorial approach: a retrospective cohort study
Source: Int J Surg. 2023 Sep 27;109(12):4062–72. doi: 10.1097/JS9.0000000000000707 (PMC10720789; doi:10.1097/JS9.0000000000000707)
Supplement: SUPPLEMENTARY MATERIAL [file js9-109-4062-s002.docx]

**
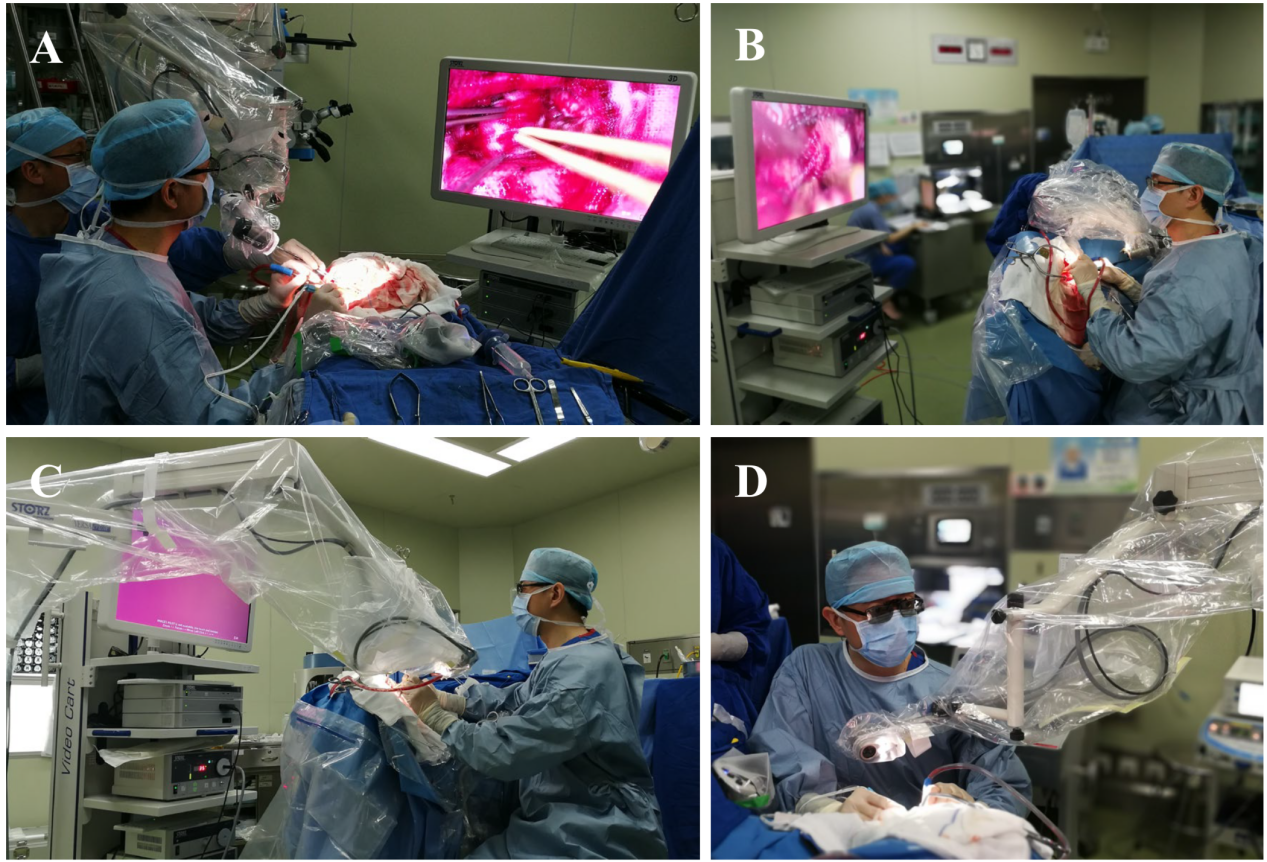
**

**Supplementary FIG. 1. A-D:** The ergonomic working scenario of the neurosurgeon during pineal region tumor resection with exoscope.


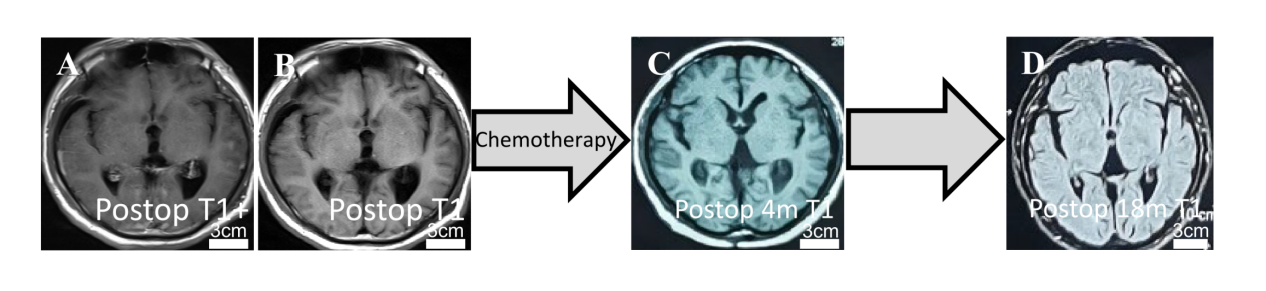


**Supplementary FIG. 2. The follow-up summary of Case 1.
A-B:** T1 contrast and T1-weighted images reveal gross total resection one week after surgery. **C:** T1-weighted image indicates no recurrence with chemotherapy 4 months after surgery. **D:** T1-weighted image shows no recurrence 18 months after surgery.
